# Novel semirigid ureterorenoscope with irrigation and vacuum suction system: introduction and initial experience for management of upper urinary calculi

**DOI:** 10.1590/S1677-5538.IBJU.2019.0521

**Published:** 2020-07-31

**Authors:** Shu Gan, Zhenlang Guo, Qianming Zou, Chiming Gu, Songtao Xiang, Siyi Li, Zhangqun Ye, Shusheng Wang

**Affiliations:** 1 The Second Affiliate Hospital of Guangzhou University of Chinese Medicine Department of Urology Guangzhou China Department of Urology, The Second Affiliate Hospital of Guangzhou University of Chinese Medicine, Guangzhou, China; 2 Huazhong University of Science and Technology Tongji Hospital Department of Urology Wuhan China Department of Urology, Tongji Hospital, Tongji Medical College, Huazhong University of Science and Technology, Wuhan, China

**Keywords:** Urinary Calculi, Vacuum, Urolithiasis

## Abstract

**Objective:**

This study aims to design a novel semirigid ureterorenoscope with irrigation and vacuum suction system and a modified ureteral access sheath (UAS) named Sotn ureterorenoscope^®^ (Sotn=ShuoTong Medical Company) to overcome the deficiencies of the current procedure and to improve the efficiency and safety of using Sotn ureterorenoscope^®^ for treatment of upper urinary calculi.

**Materials and Methods:**

Fifty-eight patients, comprising 31 males and 27 females, were evaluated. The medical records of 58 patients with upper urinary calculi treated with Sotn ureterorenoscope^®^ from March 2015 to June 2017 were retrospectively reviewed at the Second Affiliate Hospital of Guangzhou University of Chinese Medicine in China. The primary outcome was stone-free rate (SFR) assessed by computed tomography on the 1st day and one month after treatment. The secondary outcome was postoperative complication rate.

**Results:**

The mean and SD of operative duration was 48.5 (10.4) min, and the mean and SD of stone size was 15.6 (5.6) mm. The primary overall SFR was 89.7% (52/58) and 100% at 1 month follow-up. Complication, which was Clavien I (minor fever managed by antipyretic therapy), was detected in 1.7% (1/58) of the patients.

**Conclusions:**

Sotn ureterorenoscope^®^ is technically feasible, efficacious and safe for treatment of upper urinary calculi because of its advantages of high SFR and low complication rates.

## INTRODUCTION

Urolithiasis has recently attracted considerable attention worldwide because of its increasing morbidity and recurrence rates, this disease seriously affects the quality of life of afflicted individuals and increase the economic burden on the society globally (1, 2). As a low-risk procedure with a high retreatment rate (18% to 67%), extra-corporeal shock wave lithotripsy (ESWL) often leads to persistent residual stones (3, 4). When surgery is indicated for ureteral stones, ureteroscopic holmium-YAG laser lithotripsy is currently the mainstay therapy. However, the two major drawbacks of this procedure are stone retropulsion and stone fragment management in the ureter (5). Flexible ureteroscopy can minimize risks associated with bleeding and visceral injury, but the non-ideal pelvicaliceal anatomy and poor durability of flexible ureteroscopy may affect its success rate and applications (6, 7).

Percutaneous nephrolithotomy (PCNL) can be performed safely and effectively to achieve high stone-free rate (SFR) and allows for short treatment period in most patients, despite its well-known hazardous and serious complications. Most of these complications are related to tract formation and size (8, 9).

Here, we designed a novel semirigid ureterorenoscope with irrigation and vacuum suction system and its modified ureteral access sheath (UAS) named Sotn ureterorenoscope^®^ (Sotn=ShuoTong Medical Company). This study aimed to assess the efficiency and safety of using Sotn ureterorenoscope for treatment of upper urinary calculi.

## MATERIALS AND MEASURES

### Patients and methods

Our study was performed in strict accordance with the requirements of the Ethics Committee of the Second Affiliate Hospital of Guangzhou University of Chinese Medicine and under their supervision. Patients were informed that they would undergo a new technique. The risks and benefits were explained, and written informed consent was obtained from each participant or their legal guardian. Modified sheaths and specimen collection bottles were provided for free.

The inclusion criteria were as follows: (1) patients aged >18 years, (2) presence of radiopaque stones, (3) identification of upper urinary calculi and lower renal pole (upper ureteral stone, renal pelvis stone and upper and middle renal calyx stones) ≤3 cm in diameter on abdominal non-contrast computed tomography (CT) and (4) male and female patients. Patients with anatomically abnormal urinary systems (i.e. ureteral stenosis), coagulation abnormalities and uncontrolled infection of the urinary system as well as those who were pregnant were excluded from the study. SFR was evaluated according to the abdominal CT scan on the 1st day and 1st month after Sotn ureterorenoscopy. Moreover, the primary SFR was defined as the detection of residual fragments <2mm in diameter on abdominal non-contrast CT. Complications within 1 month postoperatively were assessed and classified according to the modified Clavien-Dindo classification system. Postoperative temperature of >38°C was defined as fever.

### Novel surgical device

**Figure 1:**
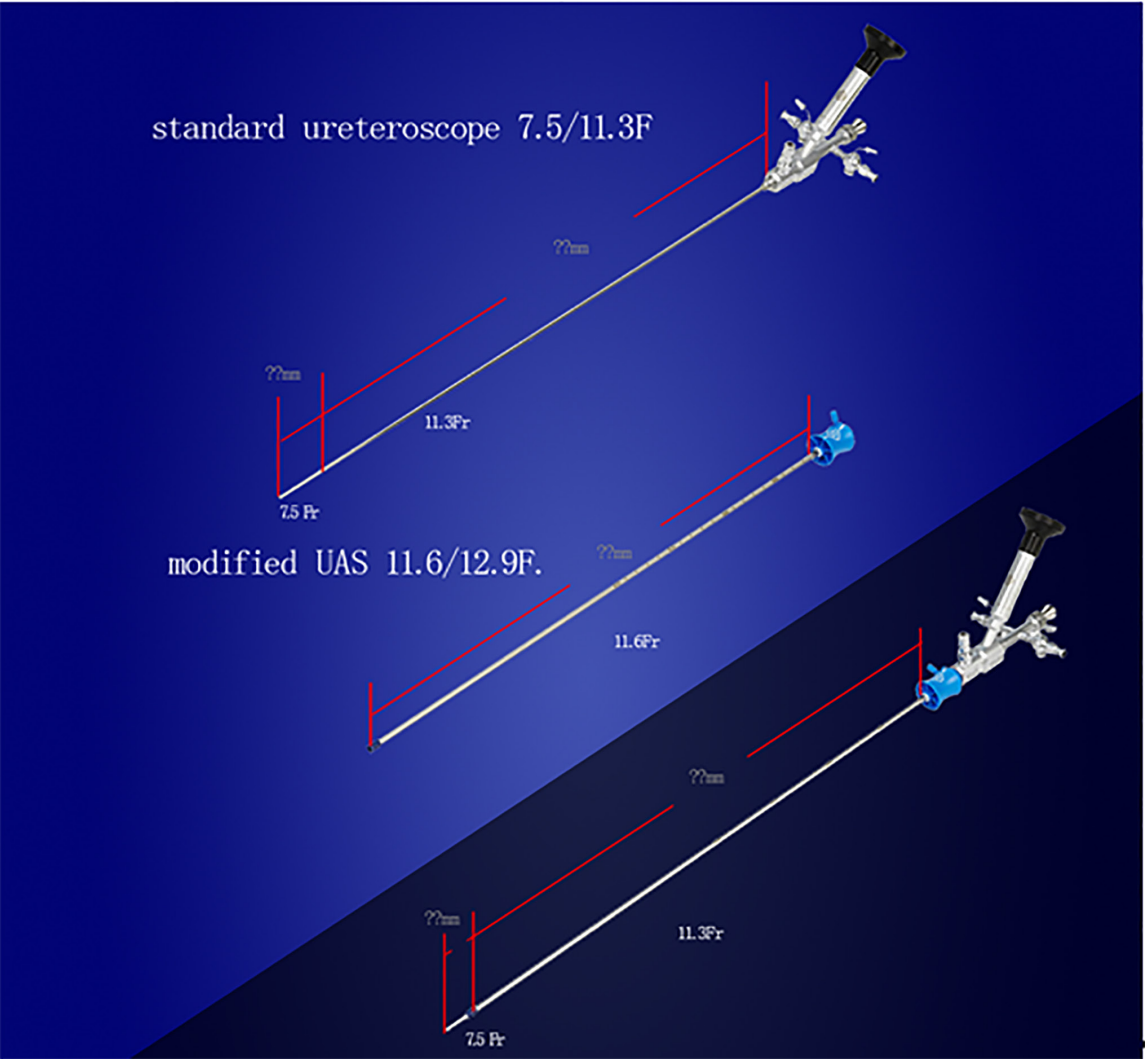
Standard ureteroscope and modified ureteral access sheath.

**Figure 2:**
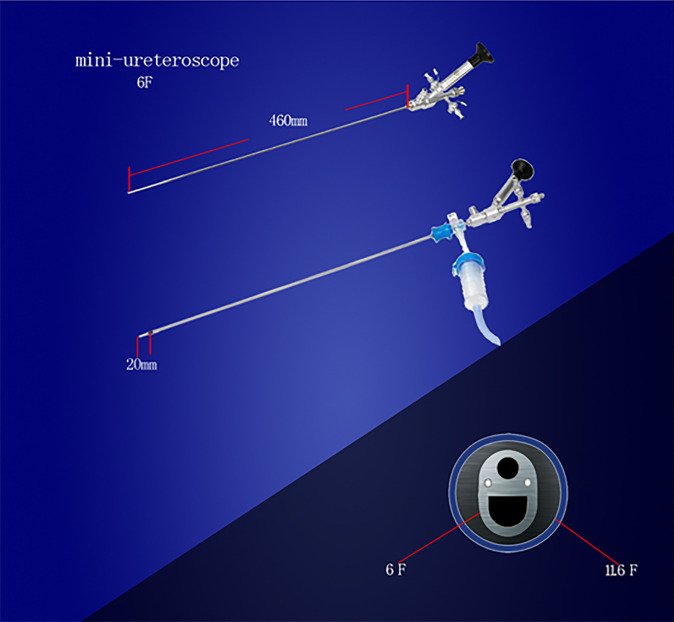
Mini-ureteroscope.

**Figure 3:**
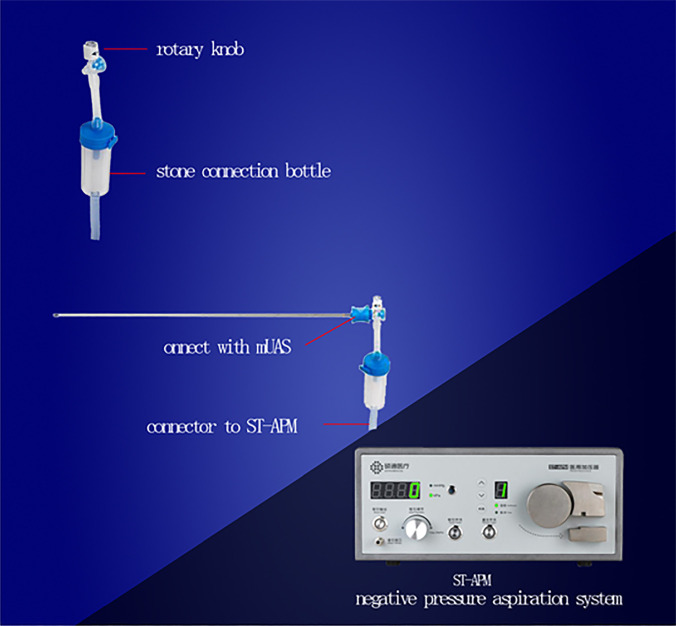
Continuous negative pressure aspiration system and Lumenis Holmium laser (maximum power, 100W).

The detailed description of Sotn ureterorenoscope^®^ is presented on the following website: http://sotnmedical.com. The new surgical equipment consists of a standard ureteroscope (length of 45cm and outer diameter of 7.5 (tip)/11.3F (shaft), Patent no. ZL201110030512.9), a modified UAS (metal material, tapered tip, no hydrophilic coating, length of 40cm and outer diameter of 11.6 or 12.9F; Figure-1; Patent no. ZL201430394938.7), mini-ureteroscope (length of 46cm and outer diameter of 4 (tip)/6F [shaft]; Figure-2; Patent no. ZL201120029461.3), an irrigation and vacuum suction system (Patent no. ZL201420607795.4), an adapter (Patent nos. ZL201430394936.8 and ZL201520891360.5) and Lumenis Holmium laser (maximum power, 100W, Figure-3).

### Surgical techniques

**Figure 4:**
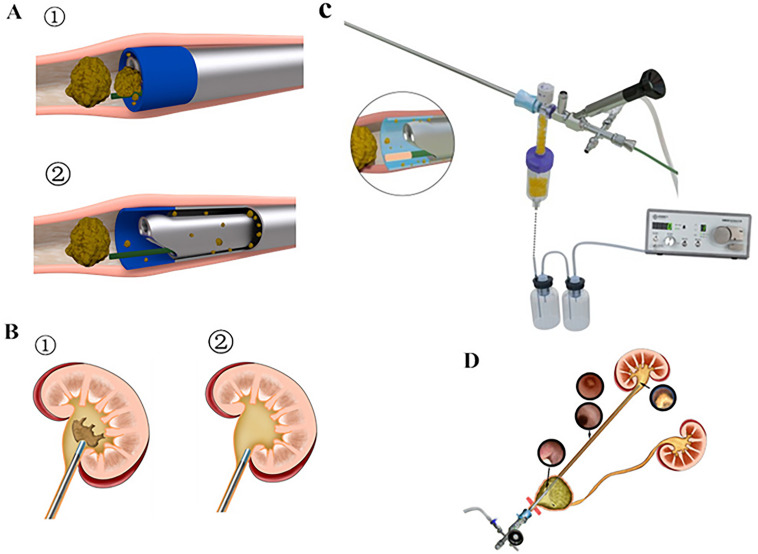
Procedures of Sotn ureterorenoscopy. A - (➀, ➁) Scheme of stone dust removal by the suctioning system through interspace between the shaft of the console ureterorenoscope and modified UAS. B - (➀, ➁) Comparison of preoperative and postoperative conditions of renal stones. C - Sotn ureterorenoscopy. D - Surgery scheme.

Patients were placed in lithotomy position, with head 30° lower and affected side 15° higher. Intratracheal intubation anaesthesia was applied in operations. The standard ureteroscope connected to the modified UAS was inserted into the upper ureter or renal pelvis guided by zebra guide wire under direct vision. The standard ureteroscope was disconnected and removed. The mini-ureteroscope was connected to the modified UAS through the adapter with stone collection bottle. The other side of the bottle was also connected to the irrigation and vacuum suction system. The 200µm laser fibre was inserted through the working channel of the mini-ureteroscope, and the stones were shredded into fragments. The laser power was set at 8-20W (0.4-1.0J, 20-40Hz). The perfusion flow speed was set in continuous mode and ranged from 60mL/ min to 610mL/min. The laser was turned on before insertion of the mini-ureteroscope with the irrigation and vacuum suction system. Negative pressure was set from-25k Pa to -4kPa in continuous mode for suction fragments, and the pressure was reduced during the operation. The renal pelvis and visible calyx were checked, and X-ray was used to confirm the absence of residual stones in the lower renal calyx during the operation. The infusion pump was stopped, and the negative-pressure suction was used when no apparent stones were found. The mini-ureteroscope and the irrigation and vacuum suction system were removed and the modified UAS with the standard ureteroscope was used. The standard ureteroscope and the modified UAS were simultaneously removed under direct vision. A 4.7F Double-J ureter stent was placed in the patient at the end of the operation and removed 2-6 weeks postoperatively. The procedure of using Sotn ureterorenoscope^®^ is shown in Figure-4, and the animated version is provided in the supplementary material (see link video).

### Statistical analysis

Statistical analysis was conducted using Stata/SE13.0. The amount data variables were described as median (interquartile range). Classification data were described as percentage. Continuous variables were assessed using Kruskal--Wallis tests for nonparametric data. Differences were considered statistically significant at P <0.05 in all tests.

## RESULTS

Fifty-eight patients, including 31 males and 27 females, were evaluated. The age of the patients ranged from 25 to 82 years, with average age of 53±12.6 years. The mean diameter of stone was 15.6±5.6mm. The mean and SD of stone volume was 1330±923mm^3^. The detailed characteristics of the patients are shown in Table-1. Among the 58 patients, the intraoperative placement of the UAS in one patient (1.7%) failed in the first stage of surgery. A 4.7F Double-J ureter stent was then placed and kept for 2-6 weeks. Operative duration was 48.5±10.4min. The overall initial SFRs were also 89.7% (52/58) and 100% at 1 month follow-up. Complication, which was Clavien I (minor fever managed by antipyretic therapy), occurred in 1.7% (1/58) of the patients, and no transfusions were needed. The post-operative renal functions of patients were also normal. No ureteral pseudochannels, perforations, avulsions, ureteric stone street formations and perirenal hematomas were detected (Table-2).

**Table 1:** Demographics and stone characteristics of patients who underwent Sotn ureterorenoscopy.

Variable		Value
Number of patients		58
Failed UAS placement of Sotn ureterorenoscopy (n, %)		1 (1.7%)
Sotn ureterorenoscopy completed (n, %)		59
Male/female (n, %)		31 (53.4%)/27 (46.6%)
Mean (SD, range) stone size (cm)		1.56 (0.56, 0.6-3.2)
**Number of stone site (n, %)**		
	Upper ureteral stone	30 (51.8%)
	Middle renal calyx stone	11 (19%)
	Renal pelvis stone	24 (41.4%)
	Multiple (ureteric and renal stones)	9 (15.5%)

**Table 2:** Intraoperative and postoperative variables.

Variable		Value
Mean (SD, range) operative time (min)		48.5±10.4
Primary SFR (n/N, %)		89.7% (52/58)
Final SFR at 1 month (n/N, %)		100%
Required auxiliary procedure (n, %)		1.7% (1/58)
Significant complication (n, %)		1.7% (1/58)
Fever (>38.5°C)		1.7% (1/58)
Blood transfusion rate		0
**Number of stone composition (n (%)**		
	Calcium oxalate stone	40 (68.97%)
	Uric acid stone	5 (8.6%)
	Calcium phosphate stone	13 (22.43%)

## DISCUSSION

Conventional option for treatment of renal stones with a maximum diameter of >20mm is open surgery or PCNL (10). PCNL is effective in treating renal stones but requires establishing channels through the renal parenchyma. Complications, including haemorrhage, infection and adjacent organ damage, were recorded when PCNL was utilized (11). Given the developments in natural endoscopic instruments and techniques, an increasing number of urological surgeons have chosen to treat renal stones by using natural channels. However, semirigid ureteroscope may be ineffective for treating upper large ureteral stones (12, 13). Flexible ureteroscope exhibits enhanced capability for treatment of all ureteral stones. Meanwhile, semirigid ureteroscope with size of <9F is a suitable device for distal ureteral calculi. When modern lithotripters are applied, approximately 90%-100% of ureteral stones can be fragmented (14). Furthermore, about 32% of patients may not be successfully treated because of mucosal oedema and ureteral stenosis. Flexible ureteroscope can be used to approach and fragmentise the located stones in such cases or stones that retropulse into the renal area (15, 16). Mursi et al. (13) reported that the SFR significantly decreased after the stones were treated with semirigid ureteroscope in the upper ureter. In a case report of 466 patients who underwent flexible ureteroscopies, 209 patients had renal stones with a maximum diameter of >20mm; the results showed that flexible ureteroscopy is safe and effective (17). In this regard, renal stones with a maximum diameter of >20mm can be safely treated using a natural channel flexible ureteroscope.

In our study, the final overall SFR was 89.7%, which was higher than that reported in a previous work on flexible ureteroscopy. A previous study comprising 316 consecutive patients who underwent flexible ureteroscopy reported an SFR of 70.5% (18). However, flexible ureteroscope with a suction system achieved primary SFR of 95.6% for patients with stone sizes ranging from 8mm to 35mm (19). This finding confirms the benefit of flexible ureteroscopy with our device. For placement of the ureter sheath, Mogilevkin et al. (20) reported that the ureter sheath cannot be used for 22% of patients in their primary surgery and should only be placed in their second surgery of flexible ureteroscopy. The ureteral wall and renal pelvis can be easily damaged because UAS is not placed by direct vision, which results in the perforation of the ureteral or pyeloneal mucosa and avulsion. Traxer et al. (21) discovered that up to 46.5% patients were injured in their ureter walls at different levels due to UAS. Finally, our study indicated that complication, which was Clavien I (minor fever managed by antipyretic therapy), occurred in 1.7% (1/58) of the patients, and no transfusions were needed. However, the intraoperative perfusion pressure during the surgery of flexible ureteroscopy was relatively high. The incidence rates of passive reflux, postoperative fever (10.7%) and sepsis (3.4%) were high (22).

Our Sotn ureterorenoscope^®^ has several important features. Firstly, the main mechanical requirements are as follows. The standard ureteroscope connected to the modified UAS was inserted into the upper ureter or renal pelvis guided by the zebra guidewire under direct vision. During the operation, the surgeon can adjust the rotary knob to control the negative pressure and actively control the pressure of the suction of stones for simultaneous reduction of the pressure inside the pelvis and active suction of the stones. The surgery was easily performed using Sotn ureterorenoscope^®^ that was improved by a ureteroscope. Lastly, our system was placed distal to the stone to fragment it. The use of suction evacuation had the advantage of removing all stone fragments without requiring a stone basket and thus shortened the operation time. Our results suggest that only one patient failed the intraoperative placement of the UAS in the first stage of surgery due to ureteral kink and stricture. We also achieved 89.7% immediate SFR and 100% SFR after 1 month in all patients.

This study indicated that the one-time success rate of the modified UAS placement was 98.6%, which is higher than the value (78%) reported by Sabnis et al. (8); the higher value in our work could be due to the fact that most medical specialists have become familiar with rigid ureteroscopy and have enriched experience. For flexible ureteroscopy, placing the modified UAS is often difficult due to uncertainty inside the ureter. Once resistance is en-countered, beginners easily fail to place the sheath. Secondly, the standard ureteroscope connected to the modified UAS was inserted into the upper ureter or renal pelvis guided by the zebra guide wire under direct vision to minimize the risk of ureteral injury. Although no damage of the renal pelvis and ureteral wall was observed, the surgeon should be careful to avoid ureter perforation and avulsion during the operation because the modified UAS of the Sotn ureterorenoscope^®^ consists of metal materials. Thirdly, active suction can decrease the pressure in the renal pelvis and reduce postoperative infection rate. The controllable negative-pressure suction system adopted by the Sotn ureterorenoscope^®^ can be controlled by the surgeon during surgery. The postoperative fever rate of this group was 1.9%, and no sepsis occurred. However, in literature, the postoperative fever rate is slightly higher (10.7%) and the rate of septicaemia is 3.4% (9). This finding confirms the various effects of the controllable negative-pressure system. When difference exists between classical surgical methods, randomised controlled studies with large sample size are needed. Finally, through active suction by negative pressure during surgery, the stone fragments can be directly suctioned in the sheath of the modified UAS. In our study, SFR values of patients in late cases were significantly higher than those in early cases and can be further improved by increasing the number of cases.

This study also displayed limitations that must be acknowledged before accepting the findings. The retrospective design employed has disadvantages regarding potential risk of bias. We plan to perform a multicentre, prospective randomised controlled trial with larger sample size in the future. The number of cases treated and evaluated with this system was reasonably low to derive certain, reliable outcomes. Finally, the developed Sotn ureterorenoscope^®^ cannot achieve real-time monitoring of the actual renal pelvic pressure and should be further improved in the future.

## CONCLUSIONS

The developed Sotn ureterorenoscope^®^ is safe, feasible and efficient for managing renal or ureter stones because of its advantages of low rate of ureteral injury, high efficacy in stone clearance, improved visual field, short operative time and ease of operation.
